# Genes involved in arsenic transformation and resistance associated with different levels of arsenic-contaminated soils

**DOI:** 10.1186/1471-2180-9-4

**Published:** 2009-01-08

**Authors:** Lin Cai, Guanghui Liu, Christopher Rensing, Gejiao Wang

**Affiliations:** 1 State Key Laboratory of Agricultural Microbiology, College of Life Science and Technology, Huazhong Agricultural University, Wuhan, 430070, PR China; 2 Department of Soil, Water and Environmental Science, The University of Arizona, Tucson, AZ 85721, USA

## Abstract

**Background:**

Arsenic is known as a toxic metalloid, which primarily exists in inorganic form [As(III) and As(V)] and can be transformed by microbial redox processes in the natural environment. As(III) is much more toxic and mobile than As(V), hence microbial arsenic redox transformation has a major impact on arsenic toxicity and mobility which can greatly influence the human health. Our main purpose was to investigate the distribution and diversity of microbial arsenite-resistant species in three different arsenic-contaminated soils, and further study the As(III) resistance levels and related functional genes of these species.

**Results:**

A total of 58 arsenite-resistant bacteria were identified from soils with three different arsenic-contaminated levels. Highly arsenite-resistant bacteria (MIC > 20 mM) were only isolated from the highly arsenic-contaminated site and belonged to *Acinetobacter*, *Agrobacterium*, *Arthrobacter*, *Comamonas*, *Rhodococcus*, *Stenotrophomonas *and *Pseudomonas*. Five arsenite-oxidizing bacteria that belonged to *Achromobacter*, *Agrobacterium *and *Pseudomonas *were identified and displayed a higher average arsenite resistance level than the non-arsenite oxidizers. 5 *aoxB *genes encoding arsenite oxidase and 51 arsenite transporter genes [18 *arsB*, 12 *ACR3*(*1*) and 21 *ACR3*(*2*)] were successfully amplified from these strains using PCR with degenerate primers. The *aoxB *genes were specific for the arsenite-oxidizing bacteria. Strains containing both an arsenite oxidase gene (*aoxB*) and an arsenite transporter gene (*ACR3 or arsB*) displayed a higher average arsenite resistance level than those possessing an arsenite transporter gene only. Horizontal transfer of *ACR3*(*2*) and *arsB *appeared to have occurred in strains that were primarily isolated from the highly arsenic-contaminated soil.

**Conclusion:**

Soils with long-term arsenic contamination may result in the evolution of highly diverse arsenite-resistant bacteria and such diversity was probably caused in part by horizontal gene transfer events. Bacteria capable of both arsenite oxidation and arsenite efflux mechanisms had an elevated arsenite resistance level.

## Background

The toxin arsenic in soil and aqueous environments is considered as one of the prominent environmental causes of cancer mortality in the World, especially in Bangladesh, India and China. In recent years, chronic intake of groundwater with high levels of arsenic has caused endemic arsenicosis in several provinces of China and new cases of arsenicosis are continuously emerging [1]. Developing efficient and environment-friendly technologies to remove arsenic from soil and water systems is of great importance to many countries including China. Bioremediation of heavy or toxic metal contaminated sites has been often shown to be more efficient than chemical and physical methods, especially when stimulating indigenous microbial communities [2].

Bacteria have developed different strategies to transform arsenic including arsenite oxidation, cytoplasmic arsenate reduction, respiratory arsenate reduction, and arsenite methylation [3]. The primary role of some of these transformations is to cope with arsenic toxicity. Arsenite-oxidizing bacteria oxidize arsenite [As(III)] to arsenate [As(V)] which in many cases is considered primarily a detoxification metabolism since As(V) is much less toxic than As(III). In addition, As(V) is negatively charged and can be easily adsorbed, thus such bacteria have been used in batch reactors together with immobilizing material for removing arsenic from waste water [4,5].

As(III) oxidation has been identified in various bacteria including *Pseudomonas *[6], *Alcaligenes *[7], *Thiomonas *[8], *Herminiimonas *[9], *Agrobacterium *[10], and *Thermus *[11]. Some of these bacteria were able to use As(III) as the sole electron donor and grew as lithotrophs. However, characterized heterotrophic arsenite-oxidizing bacteria have not been shown to gain energy through arsenite oxidation and probably use As(III) oxidation as a detoxification mechanism. Arsenite oxidation was catalyzed by a periplasmic arsenite oxidase. This enzyme contains two subunits encoded by the genes *aoxA*/*aroB*/*asoB *(small Fe-S Rieske subunit) and *aoxB*/*aroA*/*asoA *(large Mo-pterin subunit) respectively [12-14]. Recently *aoxB*-like sequences have been widely found in different arsenic contaminated soil and water systems [15].

Two families of arsenite transport proteins responsible for As(III) extrusion, ArsB and Acr3p, have been shown to confer arsenic resistance [12,16,17]. The founding member of the ArsB family, ArsB from *E. coli*, has been extensively characterized and shown to be a 45 kDa, inner membrane protein with 12 transmembrane helices [18,19]. Either ArsB alone or in association with ArsA catalyzes the extrusion of arsenite and antimonite from cells [20]. In most cases, *arsB *is co-transcribed with *arsC *encoding an arsenate reductase. It has been suggested that evolution and horizontal gene transfer (HGT) of both the ArsB and the ArsC family may have happened simultaneously in microbial evolution [12]. In many cases, As(III) is taken up by aquaglyceroporins [21] and extruded by ArsB [22].

Members of Acr3p transporters showed a function similar to ArsB, but the two proteins have no significant sequence similarity. Even though Acr3p is much less characterized, it has been reported to be present in more phylogenetically distant species than ArsB. Acr3p could be divided into two subfamilies, Acr3(1)p and Acr3(2)p, based on their phylogenetic dissimilarities [16,23]. Acr3p appeared to be more specific and transported only arsenite but not antimonite [24,25], except that Acr3p of *Synechocystis *was able to transport both arsenite and antimonite [26]. The arsenite transporter gene *arsB*, *ACR3(1) *and *ACR3(2) *have been identified in various soil bacteria [16]. However, a correlation between genotype and arsenite resistance level has not been found yet.

The impact of microbial arsenite oxidation and arsenate reduction were reported to influence environmental arsenic cycles [27]. Understanding the diversity and distribution of indigenous bacterial species in arsenic-contaminated sites could be important for improvement of arsenic bioremediation. Microbial species with arsenic biotransforming capabilities had so far not been evaluated in soil systems in China. The objectives of this study were: (1) Study the distribution and diversity of arsenite-resistant and arsenite-oxidizing bacteria in soils with different arsenic-contaminated levels; (2) Investigation of the different arsenite oxidase and arsenite transporter genes and attempt to correlate their presence to the arsenic resistance level of these bacteria.

## Results

### Distribution and diversity of arsenite-resistant bacteria in soils with different levels of arsenic

Analysis of microbial species and diversity of arsenite-resistant bacteria were performed in 4 soil samples with high (TS), intermediate (SY) and low (LY and YC) levels of arsenic contamination. A total of 230 arsenite-resistant bacteria were obtained and 14 of them showed arsenite oxidizing abilities. Based on analyses of colony morphologies and 16S rDNA-RFLP, a total of 58 strains were obtained including 5 arsenite-oxidizing bacteria.

**Figure 1:**
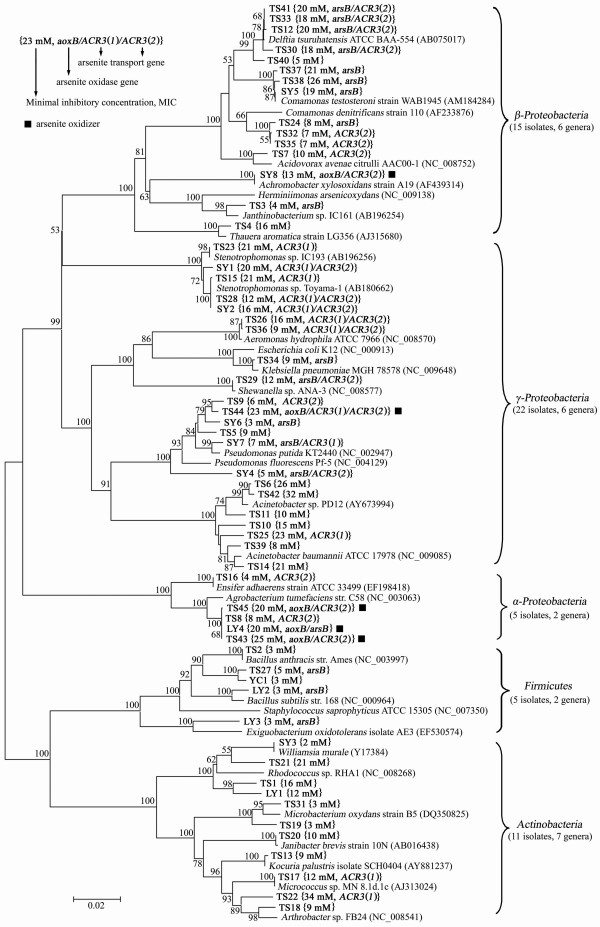
**16S rRNA phylogenetic tree, MICs, and related genes**. 16S rRNA gene (~1400 bp) phylogenetic analysis, MICs, and related genes of arsenite-resistant bacteria identified in soils with high (TS), intermediate (SY) and low (LY/YC) levels of arsenic contamination. Sequences in this study are in bold type and bootstrap values over 50% are shown. The scale bar 0.02 indicates 2% nucleotide sequence substitution.

Nearly full-length 16S rDNA sequences were used for bacterial identification. Among the analyzed 58 strains, 20 showed 100% nucleotide identities, 33 had 99% identities, 3 (*Acinetobacter *sp. TS42, *Janthinobacterium *sp. TS3, and *Delftia *sp. TS40) had 98% identities and 2 (*Acinetobacter *sp. TS11, and *Acinetobacter *sp. TS39) had 97% identities to sequences deposited in GenBank. Phylogenetic analysis divided the 58 strains into 23 genera belonging to 5 major bacterial lineages: *α-Proteobacteria *(5 strains, 2 genera), *β-Proteobacteria *(15 strains, 6 genera), *γ-Proteobacteria *(22 strains, 6 genera), *Firmicutes *(5 strains, 2 genera) and *Actinobacteria *(11 strains, 7 genera) (Fig. 1).

Among the 58 strains, 45 were isolated from the highly arsenic-contaminated soil (TS1-TS45), 8 were from the intermediate arsenic-contaminated soil (SY1-SY8) and 5 from the low arsenic-contaminated soils (LY1-LY4 and YC1) (Fig. 1). *Acinetobacter*, *Delftia*, *Arthrobacter*, *Microbacterium*, *Aeromonas*, *Acidovorax*, *Ensifer*, *Janibacter*, *Janthinobacterium*, *Klebsiella*, *Kocuria*, *Micrococcus*, *Shewanella *and *Thauera *were identified from the highly arsenic-contaminated TS soil only. *Achromobacter *and *Williamsia *were specific for the SY site. *Exiguobacterium *particularly existed in the LY/YC sites. *Comamonas*, *Pseudomonas *and *Stenotrophomonas *were identified from both the TS and SY sites. *Agrobacterium*, *Rhodococcus *and *Bacillus *were identified from both the TS and LY/YC sites. *Acinetobacter*, *Comamonas*, *Pseudomonas*, *Stenotrophomonas*, *Delftia*, *Agrobacterium *and *Bacillus *were the major arsenite-resistant bacteria in all the analyzed soil samples (37/58 = 64%).

### Arsenite-oxidizing bacteria were phylogentically distant

Five arsenite-oxidizing bacteria were identified including *Achromobacter *sp. SY8 (*β-Proteobacteria*), *Agrobacterium *spp. TS43, TS45, LY4 (*α-Proteobacteria*), and *Pseudomonas *sp. TS44 (*γ-Proteobacteria*) (Fig. 1, square black mark). All of them were heterotrophic since they could not use CO_2 _as the sole carbon source and also could not grow with arsenite as the sole electron donor (data not shown). The 16S rDNA identities of these strains were analysed and compared to the following related arsenite-oxidizing bacteria. *Achromobacter *sp. SY8 shared 98% 16S rDNA identity to *Achromobacter *sp. NT10 (GenBank accession no. AY027500) [28]. *Agrobacterium *spp. TS43, TS45, LY4 showed 99%, 98% and 99% 16S rDNA identities to *Agrobacterium *sp. 5A (GenBank accession no. AF388033) [29] respectively. The 16S rDNA identity between *Pseudomonas *sp. TS44 and *Pseudomonas putida *strain OS-5 was 97% (GenBank accession no. AY952321) [30].

### Arsenite resistance levels of arsenite-resistant bacteria vary greatly

The MIC range for arsenite of the 58 strains was from 2 mM to 34 mM (Fig. 1). The numbers of the strains with MIC values ≥ 2 mM, ≥ 5 mM, ≥10 mM, ≥15 mM, ≥ 20 mM, ≥ 25 mM and ≥ 30 mM were 58, 48, 33, 25, 17, 5 and 2 respectively. Certain correlations were found between the arsenite resistance levels, the bacterial species and soils with different arsenic-contaminated levels: (i) All of the 5 strains belonging to *Firmicutes *showed a very low MICs [*Bacillus *spp. TS2 (3 mM), TS27 (5 mM), YC1 (3 mM), LY2 (3 mM) and *Exiguobacterium *sp. LY3 (3 mM)]; (ii) Among the strains belonging to *Pseudomonas *or *Agrobacterium*, the MICs of arsenite oxidizers were higher than the non-arsenite oxidizers [*Pseudomonas *sp. TS44 (23 mM) vs *Pseudomonas *spp. TS5 (9 mM), TS9 (6 mM), SY4 (5 mM), SY6 (3 mM), SY7 (7 mM); *Agrobacterium *spp. TS43 (25 mM), TS45 (20 mM), LY4 (20 mM) vs *Agrobacterium *sp. TS8 (8 mM)]; (iii) The average MIC of the 5 arsenite oxidizers (20 mM) was higher than the 53 non-arsenite oxidizers (12 mM); (iv) A total of 12 highly arsenite-resistant bacteria [*Acinetobacter *spp. TS6, TS14, TS23 and TS42, *Arthrobacter *sp. TS22, *Comamonas *spp. TS37 and TS38, *Rhodococcus *sp. TS21, *Stenotrophomonas *spp. TS15 and TS23 and 2 arsenite oxidizers (*Agrobacterium *sp. TS43 and *Pseudomonas *sp. TS44)] whose MICs exceeded 20 mM were only obtained from highly arsenic-contaminated TS soil; (v) Only intermediate (10 mM < MICs ≤ 20 mM) and low (MICs ≤ 10 mM) MIC levels of arsenite-resistant bacteria were identified from the intermediate and low arsenic-contaminated soils; (vi) Strains of the highly arsenic-contaminated TS site had higher arsenite resistance levels (average MIC = 14 mM) than the strains of the intermediate (average MIC = 11 mM) and low arsenic soils (average MIC = 8 mM).

### Identification and distribution of the arsenite oxidase gene aoxB and arsenite transporter genes *arsB*, *ACR3(1) *and *ACR3(2)*

A total of 5 arsenite oxidase genes and 51 arsenite transporter genes were successfully amplified from 38 strains using PCR with degenerate primers. The *ACR3*(*1*) and *ACR3*(*2*) were amplified in strains of the high and intermediate arsenic-contaminated soils only. In contrast, *aoxB *and *arsB *were found in strains isolated from all three kinds of arsenic-contaminated environments (Fig. 1). Strains containing both an arsenite oxidase gene (*aoxB*) and an arsenite transporter gene (*ACR3 or arsB*) showed a higher average arsenite resistance level than the strains possessing arsenite transporter genes only [*aoxB*/*ACR3 *(20.25 mM) ≈ *aoxB*/*arsB *(20 mM) > *arsB*/*ACR3 *(14.29 mM) ≈ *ACR3 *(14.13 mM) > *arsB *(10.1 mM)].

**Figure 2:**
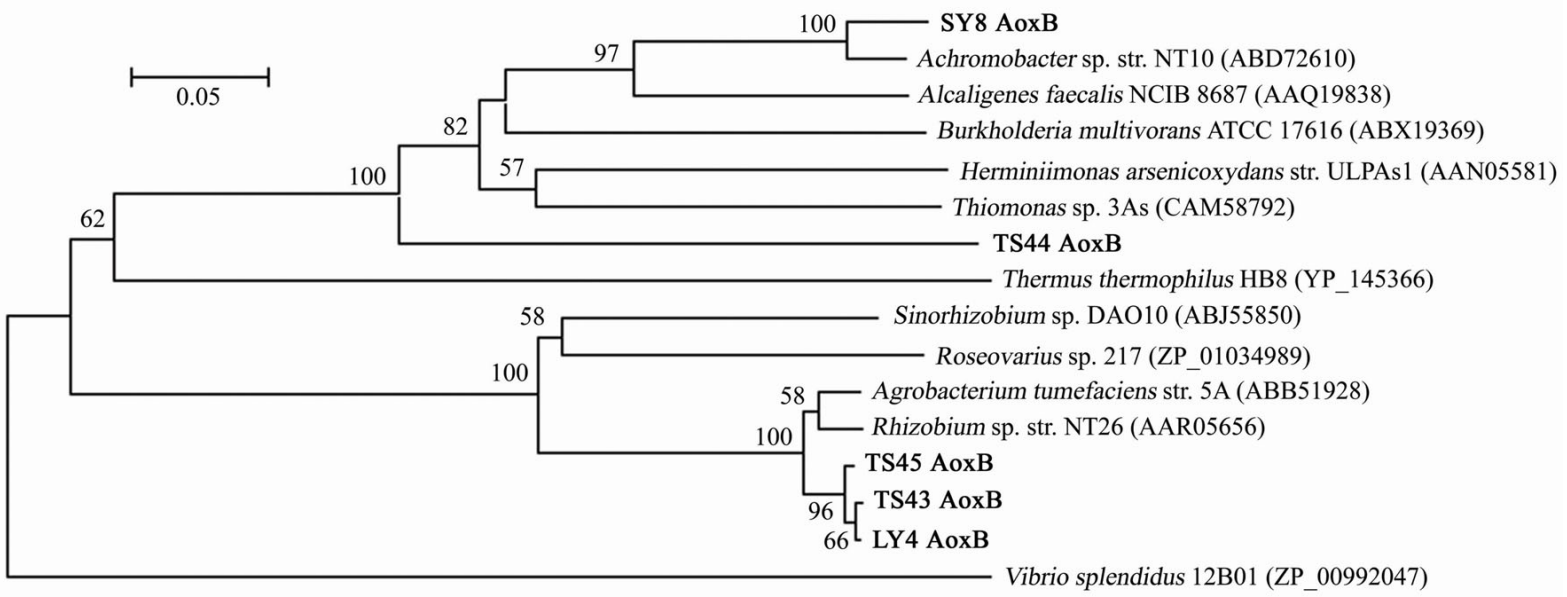
**Phylogenetic tree of arsenite oxidase (AoxB)**. Phylogenetic analysis of the deduced amino acid sequences (~160 aa) of *aoxB *genes. Sequences in this study are in bold type and bootstrap values over 50% are shown. The scale bar 0.05 means 5% aa sequence substitution.

The *aoxB *sequences were amplified from all of the five arsenite oxidizers. No *aoxB *sequences were amplified from any of the 53 non-arsenite oxidizers. The deduced amino acid sequence of *aoxB *from *Achromobacter *sp. SY8 showed 95% identity to AoxB from *Achromobacter *sp. NT-10 (GenBank accession no. ABD72610) [15]. The deduced AoxBs from *Agrobacterium *spp. TS43, TS45, and LY4 displayed 95%, 96%, and 95% identities to AoxB of *Agrobacterium tumefaciens *5A (GenBank accession no. ABB51928) [31], and 94%, 96%, and 95% identities to AoxB from *Rhizobium *sp. NT26 (GenBank accession no. AAR05656) [14] respectively. The identity of AoxBs between *Pseudomonas *sp. TS44 and *Alcaligenes faecalis *NCIB 8687 was only 61% (Fig. 2). Comparison between 16S rDNA and deduced AoxB phylogenetic trees indicated that their evolutionary relationship was similar.

**Figure 3:**
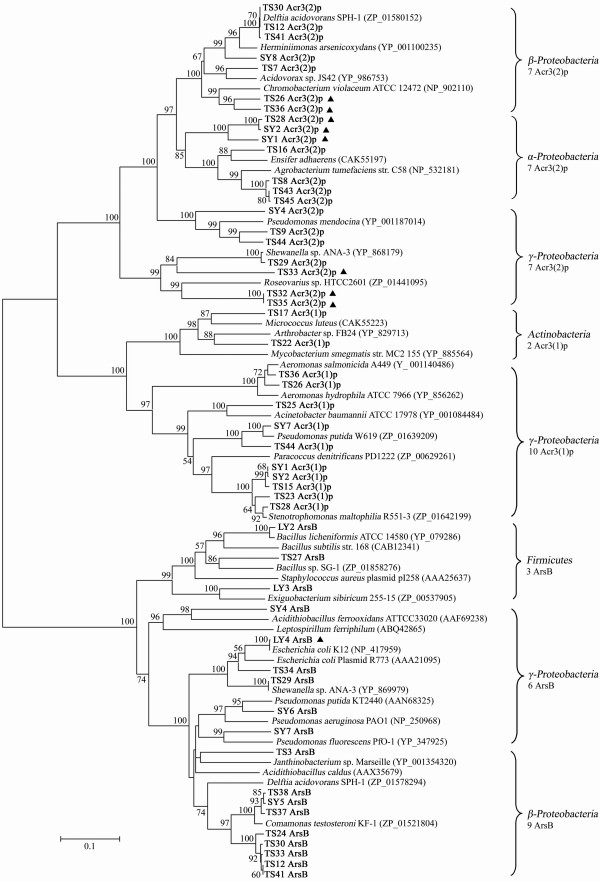
**Phylogenetic tree of arsenite transporters [ArsB/Acr3(1)p/Acr3(2)p]**. Phylogenetic analysis of the deduced amino acid sequences (~230 aa) of *arsB*/*ACR3*(*1*)/*ACR3*(*2*)genes. Filled triangles, potential horizontally transferred arsenite transporter genes. Sequences in this study are in bold type and bootstrap values over 50% are shown. The scale bar 0.1 shows 10% aa sequence substitution.

Analyses of arsenite transporter genes using the BlastX algorithm showed proteins with 71%–80% aa identities that contained 2 ArsB and 6 Acr3(2)p, 81%–90% aa identities had 10 ArsB, 5 Acr3(1)p and 11 Acr3(2)p, aa identities over 90% included 6 ArsB, 7 Acr3(1)p and 4 Acr3(2)p. The *arsB*, *ACR3*(*1*), and *ACR3*(*2*) were amplified from both the arsenite oxidizers and non-arsenite oxidizers. The strains containing 2 arsenite transporter genes simultaneously were *Pseudomonas *sp. SY7 [*arsB *and *ACR3*(*1*)], *Shewanella *sp. TS29, *Delftia *spp. TS12, TS30, TS33, TS41 and *Pseudomonas *sp. SY4 [*arsB *and *ACR3*(*2*)], *Stenotrophomonas *spp. TS28, SY1, SY2, *Aeromonas *spp. TS26, TS36 and *Pseudomonas *sp. TS44 [*ACR3*(*1*) and *ACR3*(*2*)] (Fig. 1). Phylogenetic analysis of ArsB, Acr3(1)p and Acr3(2)p showed a clear separation of the Acr3p and ArsB clusters (Fig. 3). The Acr3p cluster was further divided into two phylogenetic groups, Acr3(1)p and Acr3(2)p. The ArsB cluster was formed by 18 sequences from *β*-, *γ-Proteobacteria *and *Firmicutes*; The Acr3(1)p group had 12 sequences from *γ-Proteobacteria *and *Actinobacteria*; The Acr3(2)p group contained 21 sequences from *α*-, *β*-, and *γ-Proteobacteria *(Fig. 3).

### Horizontal transfer of arsenite transporter genes may have occurred with *ACR3(2) *and *arsB*

**Figure 4:**
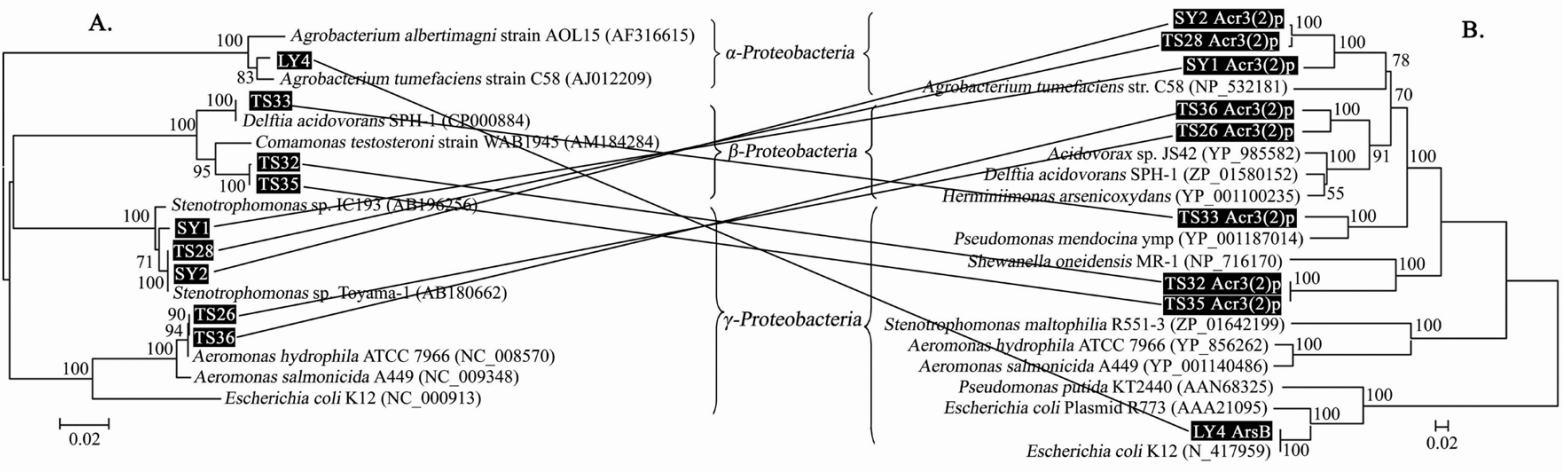
**Phylogenetic evidence of potential HGT of *arsB*/*ACR3(2)***. Phylogenetic comparison between 16S rRNA genes (A) and potential horizontally transferred *arsB*/*ACR3(2) *genes (B). All sequences used in A's and B's construction are subsets of Fig. 1 and Fig. 3 respectively.

The arsenite oxidase gene *aoxB *appeared to be vertically transferred when comparing the phylogeny of 16S rRNA genes with those encoding *aoxB*. In contrast, certain inconsistency occurred when comparing phylogenetic trees based on 16S rRNA genes and arsenite transporter genes. Phylogenetic discrepancies could be detected in 8 *ACR3*(*2*) and 1 *arsB *(Fig. 4): (i) *Aeromonas *spp. TS26, TS36 belonging to *γ-Proteobacteria *based on 16S rDNA analysis were assigned to the *β-Proteobacteria *based on Acr3p(2) sequences; (ii) *Stenotrophomonas *spp. TS28, SY2, SY1 belonging to *γ-Proteobacteria *using 16S rDNA analysis were assigned to *α-Proteobacteria *based on Acr3p(2) sequences; (iii) *Comamonas *sp. TS32, TS35 and *Delftia *sp. TS33 were shown to belong to *β-Proteobacteria*, but were assigned to the *γ-Proteobacteria *clade using Acr3(2)p sequences; (iv) LY4 belonged to *α-Proteobacteria *based on the 16S rRNA gene, but its ArsB was in *γ-Proteobacteria *clade (Fig. 4). The phylogenetic discrepancies exhibited that these 9 arsenite transporter genes were probably acquired by horizontal gene transfer (HGT). Furthermore, 6 of these horizontally transferred *ACR3*(*2*) genes were from the strains isolated from the highly arsenic-contaminated TS soil.

## Discussion

The first goal of this study was to determine the distribution and diversity of arsenite-resistant bacteria from soils with different levels of arsenic contamination. In addition, the ability to oxidize arsenite was further analyzed. Since the soils were collected from the surface and subsurface zones, only aerobic conditions were used in bacterial isolation. Thus, only aerobic/facultative aerobic bacteria were obtained in this study. We identified *Acinetobacter*, *Comamonas*, *Pseudomonas*, *Stenotrophomonas*, *Delftia*, *Agrobacterium*, and *Bacillus *as the major genera of arsenite-resistant bacteria in the four arsenic-contaminated soils. Among them, *Acinetobacter*, *Agrobacterium*, *Bacillus*, and *Pseudomonas *species were commonly found at other arsenic-contaminated sites [16,29,30,32-35]. To our knowledge, *Janibacter*, *Micrococcus*, *Thauera*, and *Williamsia *were novel arsenite-resistant bacteria isolated in this study.

We found that the high arsenic TS site revealed a much higher diversity of arsenite-resistant bacteria and the resistance levels observed were also much higher than in isolates found in the intermediate and low arsenic-contaminated sites. It is a limitation that only one medium (CDM) was used for bacterial isolation which could result in the observed differences between sites. The 12 strains with arsenite MICs > 20 mM were all obtained from the high arsenic soil. Generally, it has been proposed that high arsenic contamination is likely to exert a strong selective pressure leading to low microbial diversity [16,32]. However, the TS site used in our study had several hundred years of smelting history [36] which may result in the evolution of more bacterial species that were already well adapted at elevated arsenic concentrations. Moreover, Pennanen *et al. *[37] reported that at long-term field sites, soil microbial communities have had time to adapt to metal and/or metalloid stress. Turpeinen *et al*. [33] also found that the diversity of arsenic-resistant bacteria in higher arsenic-, chromium- and copper-contaminated soil was higher than that in less contaminated soil. These results suggested that microorganisms had been adapted to high arsenic stress and maintained their diversity in TS site after a long-term exposure to arsenic.

The *aoxB *genes were detected in all of the five arsenite oxidizers but not in the non-arsenite oxidizers. This indicates that *aoxB *may be specific for most of the aerobic arsenite-oxidizing bacteria and useful for detecting arsenite-oxidizing microorganisms in the environment. Inskeep *et al. *[15] reported that arsenite oxidase genes are widely present in different arsenite oxidizers and widespread in soil-water systems. We have enriched pristine soils with arsenite to isolate arsenite-oxidizing bacteria from non-contaminated soils but without success. To our knowledge, all of the cultured arsenite oxidizers obtained so far were isolated from arsenic-contaminated sites. Inskeep *et al. *[15] detected *aoxB*-like sequences from arsenic-contaminated environments but not from pristine soils indicating that arsenite oxidation is a major process in arsenic-contaminated environments. The expression level of *aoxB *could probably be applied to monitor environmental arsenic-contaminated levels.

A phylogenetic analysis of the 5 arsenite oxidizers based on the 16S rRNA genes and the *aoxB *genes showed a similar phylogeny indicating genomic stability of the *aoxB *genes. The MICs of the arsenite oxidizers displayed a maximal arsenite resistance level in both *Pseudomonas *and *Agrobacterium*. Furthermore, strains containing both the arsenite oxidase and any type of transporter gene showed a higher arsenite resistance level. These results suggest that bacteria capable of both arsenite oxidation and arsenite efflux mechanisms have an elevated arsenite resistance level. We also found that arsenite can be fully oxidized even at concentrations close to the MIC in arsenite oxidizers SY8 and TS44 (data not shown). Recently, we have amplified and sequenced the *arsC*/*ACR3 *operon (*arsC*_1_-*arsR*-*arsC*_2_-*ACR3*-*arsH*) in the adjacent downstream region of *aoxB *in *Pseudomonas*. sp. TS44 (data not shown; GenBank, EU311944). Kashyap *et al. *[31] found that in *Agrobacterium tumefaciens *strain 5A, disruption of *aoxR *caused a loss in the ability to oxidize arsenite and furthermore resulted in an apparent reducing phenotype probably due to the action of cytosolic ArsC and subsequent pumping out of As(III). It is noteworthy to point out that there are two processes of As(V) reduction in the environment. One is the use of As(V) as a terminal electron acceptor under anaerobic conditions. The other is the intracellular reduction of As(V) to As(III) under aerobic conditions due to the ArsC-dependent cytoplasmic arsenate reduction as part of the arsenic resistance system (*ars *operon). Since As(III) is the species being pumped out of cell (by *arsB *or *ACR3*), the presence of As(III) in the environments can also be detected under aerobic condition.

One of the main purposes in this research was to determine the correlation among the bacterial arsenite resistance level, bacterial distribution in the environment and the different types of arsenite transporter gene families. We found that the *ACR3 *genotypes were predominant over *arsB *(33 *ACR3 *vs. 18 *arsB*) in our samples which was in agreement with a report by Achour *et al*. [16]. In addition, we found any two types of arsenite transporter genes can coexist in the same strain [*arsB *and *ACR3*(*1*), *arsB *and *ACR3*(*2*), *ACR3*(*1*) and *ACR3*(*2*)]. Related reports also found the presence of multiple sets of arsenic resistance genes and operons in one strain, especially the arsenite transporter genes. *Pseudomonas putida *KT2440 contains two operon clusters (*arsRBCH*) for arsenic resistance [38]. *Acidithiobacillus caldus *has three sets of arsenic resistance determinants, one located on the chromosome and the other two exist on the transposon [39,40]. *Corynebacterium glutamicum *has two typical arsenic-resistant operons and additional *arsB *and *arsC *genes, of which two arsenite transporter genes belonged to the *ACR3(1) *group [41]. The genome of *Herminiimonas arsenicoxydans *revealed the presence of four arsenic resistance operons including two *arsB *genes and one *ACR3 *[42]. Multiple sets of arsenic resistance determinants were also reported in *B. subtilis *[18] and *Desulfovibrio desulfuricans *G20 [43]. These findings indicate that microorganisms have acquired multiple resistance determinants via chromosomal duplication or horizontal gene transfer to cope with arsenic toxicity. In this study, *ACR3*(*1*) and *ACR3*(*2*) appeared to be mostly associated with high arsenite resistance since they were only identified from the high and intermediate arsenic-contaminated sites, while *arsB *was found in all three sites. One explanation is that *ACR3 *may have a higher affinity and velocity to extrude arsenite than *arsB *and thus seems to be more effective.

Heavy metal contaminated environments were shown to provide a strong selective pressure for transfer of related resistance genes within soil systems [44]. In this study, *aoxB *and *ACR3*(*1*) appeared to be more stable than *ACR3*(*2*) and *arsB *since phylogenetic discrepancies between 16S rRNA genes and *ACR3*(*2*)/*arsB *were found which supported HGT events of *ACR3*(*2*) and *arsB*. Most of the HGT occurred in strains identified from the highly arsenic-contaminated TS soil [6 *ACR3*(*2*)]. This indicates that arsenite transporter genes may be horizontally transferred and increasingly present in a microbial population under conditions of long-term elevated arsenic stress. It is important to note that HGT occurred in somewhat closely related species in this study, however, this does not detract from the suggestion of HGT and it is likely that the HGT events occurred between these closely related species. Martinez *et al. *[45] reported that P_IB_-Type ATPases (*pbrA*/*cadA*/*zntA*) were broadly transferred in *Arthrobacter *and *Bacillus *in radionuclide and metal contaminated soils. Jackson and Dugas [46] also suggested that horizontally transferred *arsC *resulted in the diversities and complexities of arsenate reductase during its evolution. Excluding *arsC*, other genes related to arsenic resistance (e.g. *arsA*, *arsB*/*ACR3*) had not been reported as being transferred by HGT. To our knowledge, this is the first study to report widespread horizontal transfer of arsenite transporter genes. The HGT event and subsequent maintenance may have occurred increasingly under the high arsenic pressure [47] and resulted in plastic changes in microbial diversity.

## Conclusion

This work investigates the distribution and diversity of microbial arsenite-resistant species in soils representing three different levels of arsenic contamination, and further studies the arsenite resistance and arsenic transforming genes of these species. Our research provides valuable information of microbial species and genes responsible for arsenite oxidation and resistance, and increases knowledge of the diversity and distribution of the indigenous bacteria that may be stimulated for successful bioremediation of arsenic contamination.

## Methods

### Site description and soil sample collection

Four soil samples representing high (TS), intermediate (SY) and low (LY/YC) levels of arsenic contamination were used in this study. The TS soil was collected in Tieshan District, a highly arsenic-contaminated region, which is located in Huangshi City, Hubei Province, central China. There are several kinds of metal (gold, copper, iron) mines that have been smelted for many years. Concentrations of arsenic and heavy metals are extremely high in water, soil and sediments of this area [36]. The SY soil was collected from a pig farm, Shayang County, Jingmen City, Hubei Province where there are certain levels of arsenic in the soil due to the long-term usage of arsenic in feed material to resist disease and stimulate pig growth. The other two samples, LY and YC, were collected from the low arsenic-contaminated soils near the Yellow Sea of Lianyungang and Yancheng Cities Jiangsu Province, eastern China, respectively.

Several soil samples from each site were collected from the surface horizon (0–15 cm), stored at 4°C and mixed together for bacterial isolation. The total arsenic concentrations of the four soils (determined by atomic absorption spectrometry) were 337.2 mg kg^-1 ^(183.4–882.2 mg kg^-1^, SD = 184.58), 72.1 mg kg^-1 ^(43.4–94.6 mg kg^-1^, SD = 18.31), 24.1 mg kg^-1 ^(15.7–40.1, mg kg^-1^, SD = 8.24) and 34.6 mg kg^-1 ^(22.0–48.8 mg kg^-1^, SD = 8.96) for TS, SY, LY and YC, respectively.

### Isolation and identification of arsenite-resistant and arsenite-oxidizing bacteria

One hundred grams of each soil sample was amended with NaAsO_2 _to a final concentration of 500 mg kg^-1 ^and incubated at 28°C for a week. During incubation sterilized H_2_O was added to the jars to reach the original moisture value. Isolation of arsenite-resistant bacteria was performed by adding 10 g (triplicates) of each soil to 90 mL 0.85% NaCl in a 250 mL Erlenmeyer flask and shaken at 160 rpm for 30 min. 1 mL of the above mixture was added to 9 mL 0.85% NaCl for serial dilution and plated on chemically defined medium (CDM) plates [9] with a final concentration of 800 μM NaAsO_2 _and incubated at 28°C for another week. Single colonies were picked and restreaked several times to obtain pure isolates.

The obtained arsenite-resistant bacteria were tested for their abilities to oxidize As(III) (NaAsO_2_) using a qualitative KMnO_4 _screening method [10]. Each arsenite-resistant bacterium was inoculated in CDM broth with a final concentration of 800 μM NaAsO_2 _and then shaken at 160 rpm for 5 days at 28°C. For each isolate 1 mL culture was added to a 1.5 mL centrifuge tube containing 30 μL of 0.01 M KMnO_4 _and the color change of KMnO_4 _was monitored. A pink color of the mixture indicated a positive arsenite oxidation reaction [formation of As(V)]. The sterile CDM medium containing the same amount of NaAsO_2 _was used as an abiotic control. The arsenite oxidizing phenotype was also detected using the molybdene blue method with a spectrophotometer (DU800, BeckMan, CA, USA) [48].

Total DNA of each strain was extracted using standard molecular genetic methods. Nearly full-length 16S rDNA of the bacteria was amplified by PCR using universal primers Uni-27F and Uni-1492R (Table 1) [49]. PCR amplification was performed in an ATC 201 Thermal Cycler (Apollo, San Diego, CA, USA) in a 50 μL volume containing 10–50 ng DNA, 1 ng μL^-1 ^of each primer, 200 μM of each dNTP, 2.5 mM MgCl_2_, 2.5 μL dimethyl sulfoxide, 5 μL of 10 × PCR buffer [100 mM Tris-HCl (pH 8.3), 100 mM KCl] and 2.5 units of Taq DNA polymerase (Fermentas, Hanover, MD, USA), and adding ddH_2_O to a final volume of 50 μL. The PCR program consisted of an initial 5 min denaturation step at 94°C; 30 cycles of 1 min at 94°C, 1 min at 50°C, 1.5 min at 72°C; and a final extension step at 72°C for 5 min.

**Table 1:** Primers used in this study

Primer	Sequence	Reference
Uni-27F	5'-AGAGTTTGATCMTGGCTCAG-3'	
Uni-1492R	5'-GGYTACCTTGTTACGACTT-3'	49
Primers #1F	5'-GTSGGBTGYGGMTAYCABGYCTA-3'	
Primers #1R	5'-TTGTASGCBGGNCGRTTRTGRAT-3'	15
darsB1F	5'-GGTGTGGAACATCGTCTGGAAYGCNAC-3'	
darsB1R	5'-CAGGCCGTACACCACCAGRTACATNCC-3'	16
dacr1F	5'-GCCATCGGCCTGATCGTNATGATGTAYCC-3'	
dacr1R	5'-CGGCGATGGCCAGCTCYAAYTTYTT-3'	16
dacr5F	5'-TGATCTGGGTCATGATCTTCCCVATGMTGVT-3'	
dacr4R	5'-CGGCCACGGCCAGYTCRAARAARTT-3'	16

B = G, T or C; M = A or C; N = A, C, G, or T; R = A or G; S = G or C; V = A, C, or G; Y = C or T.

Colony morphologies and 16S rDNA PCR-RFLP technique were used to remove the repeated isolates for each sample. PCR-RFLP was performed by enzyme digestion at 37°C for 3 hrs in a 20 μL volume containing 2 μL of 10 × enzyme buffer, 2.5 units of *Hae*III or *Msp*I and 5–10 μL of the 16S rDNA PCR products, amending ddH_2_O to a final volume of 20 μL. The digested DNA fragments were separated in 2% agarose gels and the digestion patterns were grouped by DNA fingerprinting profiles.

### Identification of the *aoxB *gene encoding the arsenite oxidase Mo-pterin subunit and *arsB*, *ACR3(1) *and *ACR3(2) *genes encoding different arsenite transport proteins

The PCR amplification of *aoxB *was performed using degenerate primers (Primers #1F and #1R) (Table 1) and following the PCR conditions as described by Inskeep *et al*. [15]. The amplification of *arsB*, *ACR3*(*1*) and *ACR3*(*2*) genes were performed using three pairs of degenerate primers [darsB1F and darsB1R for *arsB*, dacr1F and dacr1R for *ACR3*(*1*), dacr5F and dacr4R for *ACR3*(*2*)] (Table 1) as described by Achour *et al*. [16]. The PCR products were purified using the Gel Extraction Kit (SBS Genetech, Shanghai, China). The purified PCR products were ligated into pGEM-T (Promega, Madison, WI, USA) and the ligation products were used to transform *E. coli *DH5*α *competent cells by electroporation. The transformants were grown on LB agar containing ampicillin, X-Gal and IPTG at 37°C for 16 hrs according to the manufacturer's recommendations.

### DNA sequencing and phylogenetic analysis

The PCR products were purified using the UltraPure™ PCR Kit (SBS Genetech). DNA sequencing analysis was performed using ABI 3730XL DNA analyzer by Sunbiotech company (Beijing, China). All sequences were analyzed by BlastN (for 16S rRNA gene) and BlastX (for deduced AoxB and ArsB/Acr3p) searching tools [50]. All sequences were checked manually and edited for the same lengths using ClustalX 1.83 software [51]. MEGA 3.1 software [52] was used to construct phylogenetic trees by the neighbor-joining distance method [53] and reliability of inferred trees was tested with 1000 bootstrap replicates. Some reference sequences from the GenBank were used in constructing phylogenetic trees for clarification.

### Determination of the minimal inhibitory concentrations (MICs) of arsenite

The MIC, defined as the lowest concentration of arsenite that inhibited growth in CDM broth, was performed with all arsenite-resistant bacteria. Triplicate samples of each single colony were inoculated in 3 mL CDM broth supplemented with increasing concentrations of NaAsO_2_, incubated with shaking at 28°C for one week and the OD_600 _values were determined. The initial screening for MICs was performed with 5 mM, 10 mM, 15 mM, and 20 mM of NaAsO_2_. Subsequent determinations were performed with 1 mM NaAsO_2 _intervals over the appropriate range. The sensitivity of MIC detection was 1 mM.

### Nucleotide sequence accession numbers

The nucleotide sequences are posted in the NCBI GenBank database. Their accession numbers are: EU073067–EU073124 for 16S rRNA genes, EF523515, EU311944–EU311947 for *aoxB*, and EU311948–EU311999 for *arsB/ACR3*.

## Authors' contributions

All authors participated in the design of the study and data analyses. LC carried out samples collection, bacterial isolation and drafted the manuscript, participated in molecular genetic studies. GL carried out molecular genetic studies and construction of phylogenetic trees. CR participated in the design of the experiments and helped to draft the manuscript. GW is the Principal Investigator of the funded projects. She coordinated the study and helped to draft the manuscript. All authors read and approved the final manuscript.
